# Kinematic and Electromyographic Activity Changes during Back Squat with Submaximal and Maximal Loading

**DOI:** 10.1155/2017/9084725

**Published:** 2017-05-04

**Authors:** Hasan U. Yavuz, Deniz Erdag

**Affiliations:** ^1^Department of Sports Medicine, Faculty of Medicine, Near East University, Nicosia, Northern Cyprus, Mersin 10, Turkey; ^2^School of Physical Education and Sport, Near East University, Nicosia, Northern Cyprus, Mersin 10, Turkey

## Abstract

The aim of this study was to investigate the possible kinematic and muscular activity changes with maximal loading during squat maneuver. Fourteen healthy male individuals, who were experienced at performing squats, participated in this study. Each subject performed squats with 80%, 90%, and 100% of the previously established 1 repetition maximum (1RM). Electromyographic (EMG) activities were measured for the vastus lateralis, vastus medialis, rectus femoris, semitendinosus, biceps femoris, gluteus maximus, and erector spinae by using an 8-channel dual-mode portable EMG and physiological signal data acquisition system (Myomonitor IV, Delsys Inc., Boston, MA, USA). Kinematical data were analyzed by using saSuite 2D kinematical analysis program. Data were analyzed with repeated measures analysis of variance (*p* < 0.05). Overall muscle activities increased with increasing loads, but significant increases were seen only for vastus medialis and gluteus maximus during 90% and 100% of 1RM compared to 80% while there was no significant difference between 90% and 100% for any muscle. The movement pattern in the hip joint changed with an increase in forward lean during maximal loading. Results may suggest that maximal loading during squat may not be necessary for focusing on knee extensor improvement and may increase the lumbar injury risk.

## 1. Introduction

Squat is one of the most frequently used exercises in many training protocols. Due to its applicability to functional exercise and sports, numerous variations have been developed and employed in the fields of strength and conditioning and physical therapy [1]. Since it has biomechanical and neuromuscular similarities to a wide range of athletic movements, it is a core exercise in many sport routines [2, 3].

The purpose of squat is to train the muscles around the knees and hip joints, as well as to develop strength in the lower back, for execution of basic skills required in many sporting events and activities of daily living [4].

The athlete takes an initial stance position with heels approximately shoulder-width apart and toes pointing forward or slightly outward by no more than 10 degrees [5]. Squat maneuver begins with the lifter in an upright position, knees and hips fully extended. The lifter then squats down by flexing at the hip, knee, and ankle joints. When the desired squat depth is achieved, the lifter ascends back to the upright position [2]. The lumbar vertebrae are maintained in a neutral alignment throughout the entire squat movement, and the trunk should remain as upright as possible during squat [6]. The athlete's feet should be stable and planted firmly on the ground and the athlete should keep his entire foot on the ground throughout the entire squat motion. At the proper depth, the femurs are slightly past parallel to the ground, the hips are flexed, the tibias are positioned vertically, and the feet are entirely on the ground [5].

As a multijoint exercise, the knee extensors (e.g., rectus femoris (RF), vastus lateralis (VL), and vastus medialis (VM)) and the hip extensors (e.g., gluteus maximus (GM), biceps femoris (BF), and semitendinosus (ST)) are considered to be the prime movers during squat exercise, with other muscles acting in a secondary capacity [7–9].

Activation of specific muscles is important in designing a workout that effectively utilizes targeted muscles so that time and effort are not wasted by performing exercises that may not provide the desired benefits. To be able to get the maximum benefit from the exercise, it is very important to perform the proper technique with a well-designed set and repetition numbers. It is a well-known fact that the number of repetitions decreases when the load increases [10–12]. Although it is advised that the technique should remain the same as the exercise intensity is increased [5], it is a common observation for many trainers that when the loads reach to the limits of the athletes, athletes may change the movement pattern of the performed exercise. These manipulations of the exercises may help the athletes to be able to lift higher loads. This generally happens during maximum loading (1RM) or lasts a few repetitions of the maximum number of repetitions. The 1 repetition maximum (1RM), which is the heaviest load that can be lifted once during a traditional weightlifting-type task, is an isoinertial measure that gives an estimate of maximum strength. Regardless of which aspect of strength the coach or athlete decides is appropriate for a given sport, maximum strength should first be developed because it acts as a general base that supports specific training in other spheres of conditioning [13].

The term “cheating” is mostly used for these biomechanical manipulations. Cheating may cause changes in kinematics of the specific exercise. Athletes who do not demonstrate proper mechanics may utilize compensatory movement strategies that can hinder their athletic performance and heighten their risk of sports-related injury [5, 14, 15]. In this case, the proper technique may be more important than the load not only for preventing the injuries but also for muscle activity.

Although squat is a closed kinetic chain exercise, the athletes may change the movement pattern and therefore muscle activities during maximal loading.

There are many studies that analyzed a variety of biomechanical variables during squat to provide athletes and their coaches with information to guide training prescription, progression, and technique instruction [16–19], but the impact of maximal loading on kinematic patterns has been largely unexplored. Surface EMG has been widely used for assessment of muscle activities during squat, and it has been shown that the EMG activities of the muscles increased by increasing the loads during squatting in different studies [20–23]. Bryanton et al. [24] studied the biomechanical analysis and relative muscular effort during squat while increasing the barbell load from 50% to 90% of 1RM but did not use EMG to determine the muscle activities and more importantly the maximal loading that we expect to see the kinetic and muscular activity changes. Therefore, we aimed to see the kinematic and muscular activity changes while we increase the loads from 80% to 90% and finally 100% of 1RM squatting. To our knowledge, this is the first study to determine the kinematic and EMG activity changes while increasing the loads from submaximal to maximal.

## 2. Materials and Methods

### 2.1. Subjects

Fourteen healthy male recreational bodybuilders (21.6 ± 2.3 years old), who were experienced at performing squats (3.2 ± 0.6 years), participated in this study. The average height and weight of the participants were 178.4 ± 5.1 cm and 80.1 ± 7.2 kg, respectively. All subjects were right handed and had no history of orthopaedic injury or surgery that would have limited their ability to perform the squatting techniques.

The mean 1RM loads that were employed during testing were 120.0 ± 24.2 kg, and this was 150.4 ± 32.5% of their body weight.

Before participation, informed consent was obtained from each subject. The investigation was conducted according to the Declaration of Helsinki and approved by the Near East University Scientific Researches, Evaluation and Ethic Commission (YDÜ/2012/11-60).

### 2.2. Instrumentation

We followed the methods of Yavuz et al. [25], an 8-channel dual-mode portable EMG and physiological signal data acquisition system (Myomonitor IV, Delsys Inc., Boston, MA, USA) was used for data collection. Data collections were conducted using EMG Works Acquisition 4.0.5 (Delsys Inc., Boston, MA, USA). The amplifier bandwidth frequency ranged from 20 to 450 Hz, with an input voltage of 9 VDC at 0.7 A and the common-mode rejection ratio was 80 dB. Data were recorded at a sampling rate of 1000 Hz over a wireless local area network (WLAN) to the host computer for real-time display and storage [25].

Seven channels of this system were used to assess the EMG activity of vastus lateralis (VL), vastus medialis (VM), rectus femoris (RF), semitendinosus (ST), biceps femoris (BF), gluteus maximus (GM), and erector spine (ES). Recording sites were prepared by shaving the area and wiping with alcohol pads to decrease electrical impedance. Electrodes (41 × 20 × 5 mm, D.E 2.3, Delsys Inc., Boston, MA) were placed along the longitudinal axis of each muscle tested on the right side (dominant side) of the participant's body according to the procedures from Gullet et al. [4] (Table 1). The sensor contacts are made from 99.9% pure silver bars measuring 10 mm in length, 1 mm in diameter, and spaced 10 mm apart from optimal signal detection and consistency. A 5.08 cm diameter oval-shaped common reference electrode (Dermatrode HE-R, American Imex, Irvine, CA) was placed on the iliac crest of the right leg. Positions of each electrode in relation to the muscle being tested are shown in Table 1 [25].

At the same time, the EMG system was synchronized with a Samsung (VP-D375W) video camera with a shutter speed of 1/250 seconds and frame rate at 25 frames per second by using the National Instruments USB-6501 Digital I/O trigger box (Delsys Inc., Boston, MA, USA). The frame rate was then increased to 50 frames per second by using the deinterlace method. Video recordings were made with AMCap (Microsoft, V 3.0.9) video capture software. For kinematical data, reflective markers (3 cm diameter) were attached and positioned over the following bony landmarks: (a) lateral malleolus of the right foot, (b) upper edges of the lateral tibial plateau of the right knee, (c) posterior aspect of the greater trochanters of the right femur, and (d) end of the right side of the Olympic bar. A calibration plane that consists of 8 control points was used for 2D spatial reconstruction.

A standard 20.5 kg Olympic barbell, discs (Werksan, Turkey), and a continental squat rack were used during the squat [25].

### 2.3. Exercise Protocol

The subjects were required to attend two sessions. A pretest was given to each subject 1 week before the actual testing session. The experimental protocol was reviewed, and the subjects were given the opportunity to ask questions. During the pretest, the subject's 1RM was determined and recorded. The procedure used for assessing 1RM was described by Kraemer and Fry [27]. The subjects were asked to perform initial preparation on a stationary bike for 3–5 minutes at the beginning of the pretest session and then to perform a warm-up set of 8–10 repetitions at a light weight (approximately 50% of estimated 1RM). A second initial preparation consisting of a set of 3–5 repetitions with moderate weight (approximately 75% of 1RM) and third initial preparation including 1–3 repetitions with a heavy weight (approximately 90% of 1RM) followed.

After a 5-minute rest, participants performed 1 trial of squats with the load (~80% of estimated 1RM) through the full range of motion. After each successful performance, the weight increased until a failed attempt occurred. Squat trials were considered successful if the participant reached 90° of knee flexion, which was marked by adjustable stoppers, returned to the starting position, and maintained the metronome cadence (40 beats per minute Largo). Five-minute rests were given between each attempt and the 1RM was attained within 5 attempts and a 5-minute rest separated each test.

Subsequently, 1 week after the pretest session, the subjects were asked for a second session for data collection.

After the EMG electrode placement, maximum voluntary isometric contraction (MVIC) data from the quadriceps, hamstrings, erector spine, and gluteus maximus were collected according to the procedures described by Konrad [28]. Three 3-second MVIC trials were collected in a randomized manner for each muscle group. Adequate rest was allowed between trials (1 minute).

All subjects performed two to three warm-up sets in preparation for testing. Then they performed squats with 80%, 90%, and 100% of the previously established 1RM with a 5-minute rest between trials. Exercise began with a given verbal command. The starting and ending positions for back squat were performed with the legs at shoulder width apart and the toes pointed forward, feet are entirely on the ground with the knees in full extension, which was defined as a 180° knee angle (KA). From the starting position, the subject flexed their knees to minimum KA (approximately 90°) and then extended their knees back to the starting position. A metronome cadence (40 beats per minute Largo) was used to control movement speed between the participants.

### 2.4. Data Reduction

The samplings of EMG and video recordings were initiated simultaneously with the beginning of the first squat repetition. For synchronization, a LED connected to the trigger box (National Instruments USB-6501 Digital I/O) outputted a digital signal when the Myomonitor started data acquisition. With the data acquisition, the LED lit, and it went off as the data stopped to show the exact start and stop moments of collected data for the synchronization with the kinematics.

Kinematical data were analyzed by using the saSuite 2D kinematical analysis program, which was developed by Hacettepe University, Faculty of Sports Sciences, Biomechanics Research Group, Ankara, Turkey. For each trial, required portions of the video recordings were trimmed, the anthropometric points were digitized, and 2D positional data were obtained. To analyze angular kinematics of the knee and hip joints, the raw data points were calculated and then smoothed using a moving average filter [29]. All EMG data were partitioned into ascending and descending phases. The time from the initiation of the flexion of the hips and knees until the greater trochanter reached its lowest point defined the descending phase of each repetition of squat. The ascending phase followed the descending phase and consisted of knee and hip extensions from the parallel thigh position until the subject was standing erect at the end of the repetition [4]. Movement time was normalized by using metronome cadence (1.5 seconds for descending and 1.5 seconds for ascending phases, 40 beats per minute Largo) and divided into ten movement phases to control interindividual differences. Knee and hip angle (between the thigh and trunk) changes were examined throughout the descending and ascending phases.

EMG data were analyzed according to the procedures from the International Society of Electrophysiology and Kinesiology [30] by using the EMG Works Analysis 4.0 (Delsys Inc., Boston, MA, USA). To calculate the mean-normalized EMG values, the raw EMG signals were subsetted, filtered (passband: 3, response: band pass, corner F1: 10 Hz, corner F2: 500 Hz), rectified, integrated (root mean square (window length: 0.100, window overlap: 0.08, remove offset)), and normalized to the subject's highest corresponding MVIC trial.

### 2.5. Statistical Analysis

Kinematic and electromyographic data were analyzed and compared with *repeated measures analysis of variance* (*p* < 0.05). Thru out the text, data for all subjects performing each type of exercise were averaged and presented as means and standard deviations.

## 3. Results

**Figure 1:**
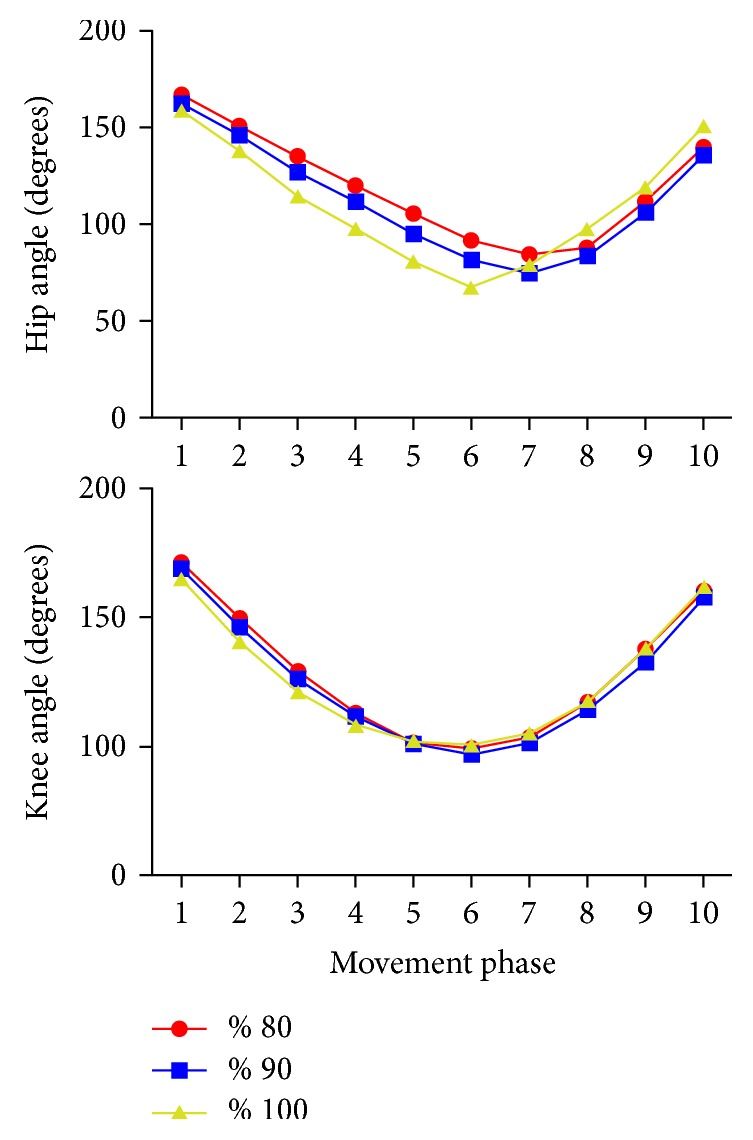
Mean knee and hip joint angles throughout the descending and ascending phases of the squat movement.

Mean knee and hip joint angles throughout squat movement in different loads were presented in Figure 1(). Although the similar movement patterns in the hip joints with 80% and 90% loads, the hip angles seemed decreased with 90% loads especially during the descending phase. The decrease in hip angles was more obvious with maximum loading (100%) and there seemed to be an increase in hip angles during the ascending phase. What is interesting in this data is that the ascend also started earlier in the hip angle with 100% loads.

Table 2 presents the mean (±SD) repeated measures analysis of variance results of the normalized EMG values of seven muscles tested during back squats throughout the whole squat motion. The EMG values increased with the increase of the load but revealed no significant differences (*p* < 0.05) except for VM and GM. For the VM and GM, 90% and 100% bar loads of 1RM, respectively, were found greater than 80% loads (*p* < 0.05). There was no statistically significant difference between 90% and 100% loads for any muscle activity.

Table 3 presents the mean (±SD) repeated measures analysis of variance results between 80, 90, and 100% loads of 1RM during the descending and ascending phases. Results showed that the EMG muscle activity values were increased with the increase of the load both for descending and for ascending phases. Moreover, 90% VM, ES, and GM descending phase, 100% ES and GM descending phase, 100% VM and GM ascending phase muscle activity EMG values were found significantly different compared to that of 80% loads of 1RM (*p* < 0.05). No significant difference was observed for any muscle neither during ascending nor during descending phases between 90% and 100% loads.

**Figure 2:**
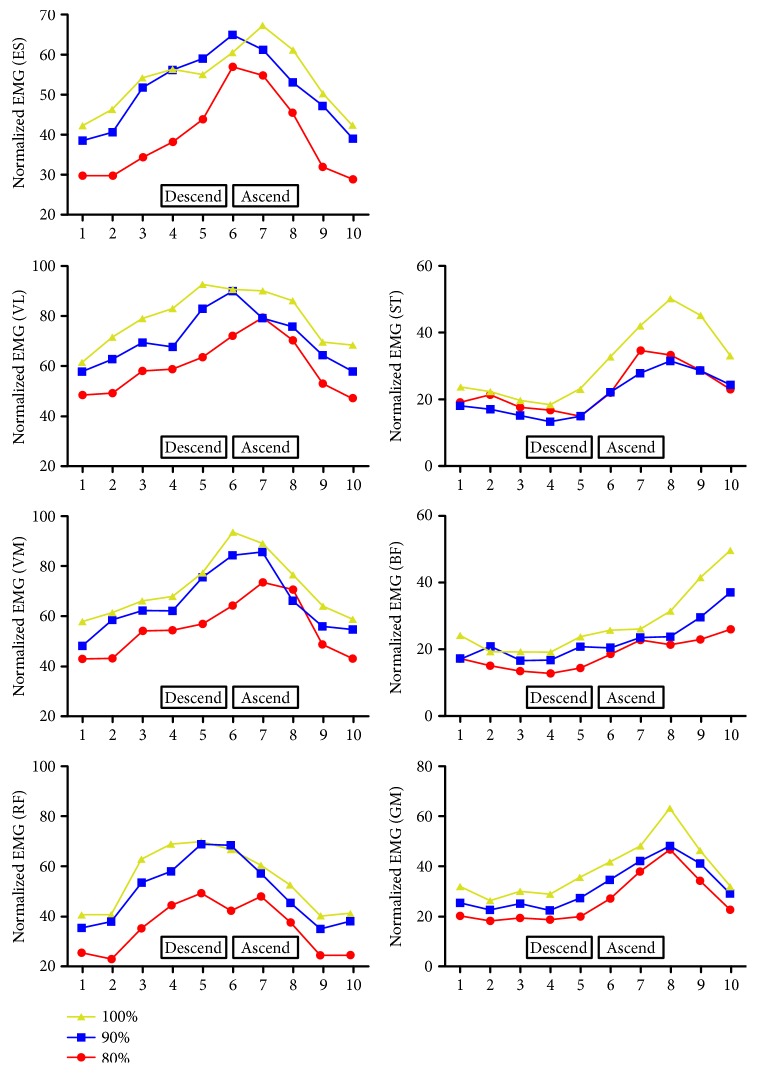
Knee and hip angle-dependent EMG values of rectus femoris (RF), vastus medialis (VM), vastus lateralis (VL), erector spinae (ES), gluteus maximus (GM), biceps femoris (BF), and semitendinosus (ST) throughout the descending and ascending phases of the squat movement.

Figure 2 illustrates knee and hip angle-dependent EMG values of the muscles throughout the phases of squat movement.

## 4. Discussion

The present study was designed to investigate the possible kinematic and muscular activity changes during squat maneuver while the loads are increased from 80% to 90%, and finally 100% of 1RM squatting. We aimed to see how the athletes manage to lift maximum loads by increasing the muscular activity or changing the movement pattern or both.

A strong relationship between load and muscle average EMG activity has been reported in the literature [20–23, 31], and the result of this study was in accordance with this literature. Although the overall increase in muscle activities corresponds with increasing loads, significant increases were seen only for VM and GM during 90% and 100% of 1RM, respectively, compared to those during 80% while there was no statistically significant difference between 90% and 100% for any muscle. But when we divide the maneuver to descending and ascending phases, we also saw an increase in the ES EMG activities during the descending phase with 90% and 100% loadings. Several studies have shown that manipulating features of the squat exercise resulted in altered muscle activity. These manipulations include changes in foot position [20, 22], barbell position [4], stability of the surface on which the exercise is performed [32–34], different levels of intensity of load [23], range of motion [7, 23, 35], and different equipment [36].

**Figure 3:**
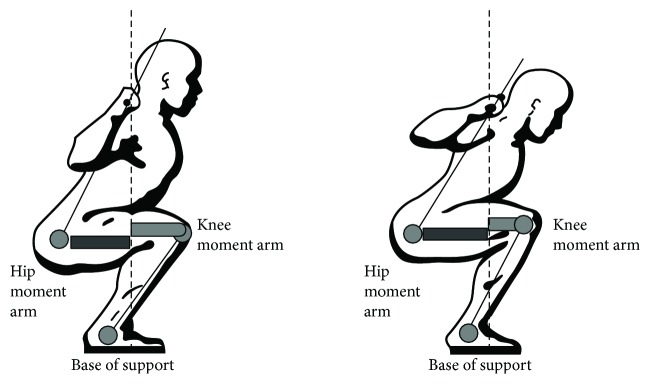
Knee and hip moment arms during squat with different hip angles (modified from starting strength: basic barbell training, by permission of The Aasgaard Company, Rippetoe and Kilgore [37]).

Aspe and Swinton [23] indicated that the heavier external resistance during squat creates a larger resistance moment, which requires greater muscular effort to counterbalance. The increased forward lean would also create a larger resistance moment by lengthening the hip moment arm to counterbalance much harder during descend (Figure 3). That might be a reason for increased ES EMG activities during the descending phase of the heavier loads.

In our previous study, where we compared front and back squat maneuvers, an increased forward lean was observed during back squat compared to that during front squat [25]. We found greater VM EMG activities during front squat and greater ST activities during back squat. However, we did not see an increase in ST activities despite the similar increase in forward lean in the current study. However, in our previous study, we compared two different squat techniques (front and back squat) with similar loads (100% of 1RM) while we compared different loads (80%, 90%, and 100% of 1RM) during the same squat technique (back squat) in the current study. So, both different techniques and/or different loads may be the reason for different muscle activity changes despite the increase in forward lean.

In addition to increased forward lean, a change in the movement pattern of the hip joint was observed during 100% loading. The ascend started with knee extension in the 6th phase and followed by hip extension in the 7th phase during 80% and 90% loading while hip extension starts earlier (in the 6th phase) during 100% loading (Figure 1). That means that the initial moment required to start upright movement was created not only by the knee extensors but also by the hip extensors. This may also be supported by EMG activities. GM EMG activity shows a significant increase only in the descending phase to help in counterbalancing during 90% loading while it shows a significant increase in both descending and ascending phases during 100% loading (Table 3). The common faults in the early stages of learning back squat (i.e., early training age) are for the hips to rise faster than the shoulders, which would increase trunk flexion. If the hips rise too quickly, the vertical distance between the hips and shoulders will decrease during the early ascent phase. Irrespective of the load, the movement pattern represents an incorrect back squat that can be a dangerous strategy to the lower back during squatting with progressive external resistance [5].

Another interesting finding was that the forward lean increased during the descending phase but decreased during the ascending phase with 100% loading (Figure 1). When the minimum knee angle is arrived, the knee and hip joint starts to extend. A more upright position can be provided by hip extensor activity and creates a shorter hip moment arm and a longer knee moment arm that will help the knee extensors to do more work. That may mean more active contribution of the hip joint to the movement during 100% loading. Although it may help the athletes to lift higher loads when the center of gravity moves further away from the lumbar spine increasing the moment arm and torque, the shear forces occurring within the lumbar spine would also increase [4, 38, 39] (Figure 3). Increased forward lean reduces tolerance to compressive load and results in a transfer of the load from muscles to passive tissues, heightening the risk of disc herniation [40].

It is beneficial to maintain a posture that is as close to upright as possible at all times for preventing the lumbar injuries [8]. The increased movement of the hip joint thereby the center of gravity may increase the risk of lumbar injury. This “knee-loading” strategy instead of “hip-hinge” strategy that can be seen with excessive trunk flexion may also place excessive shear forces on the anterior knee and decreases recruitment of the posterior chain musculature [5]. That may increase the risk of knee injuries.

When we check angle-specific EMG changes for each muscle, we saw that EMG activity patterns were quite parallel to each other for most of the muscles in all loads. But earlier peak EMG activities were seen for knee extensors, and this may also be because of the earlier contribution of hip extension to the maneuver.

It is known that the hamstrings (biceps femoris, semitendinosus, and semimembranosus) are technically antagonists of the quadriceps, opposing knee extensor moments. However, in squat, a closed chain exercise, they behave paradoxically and cocontract with the quadriceps. In this study, hamstring EMG muscle activity was found increased as the loads increased with the biceps femoris producing more EMG muscle activity than the semitendinosus. These results are consistent with the other studies [2, 3, 41].

It is advised that athletes do not increase the intensity of squat (i.e., increase resistance) unless the athlete can demonstrate a consistent, proper form of back squat. The technique should remain the same as the exercise intensity is increased [5].

## 5. Conclusion

We studied musculature activity and kinematics of the knee and hip joints during squat with 80%, 90%, and 100% of maximum loading (1RM). The EMG activity for all muscles increased with increased load but only statistically significant differences were found for VM and GM. There was no statistically significant difference for any observed muscle activity between 90% and 100% of 1RM. Hip joint kinematics showed a different movement pattern for 100% loading.

The 90% loading was shown to be just as effective as the 100% loading conditions in terms of overall muscle activity, with no difference in knee joint kinematics and less contribution of the hip joint, which may probably show less lumbar and knee injury risks. Results may suggest that 100% loading during squat may not be necessary to focus on knee extensor improvement. It may be a better choice to use the equipment that may prevent changing the movement pattern for preventing lumbar injuries during maximal loading.

## Conflicts of Interest

The authors declare that they have no conflicts of interest.

## Figures and Tables

**Table 1:** A description of the positioning of each electrode in relation to the muscle being tested developed by Brouer and Houtz [26] and described by Gullet et al. [4].

Muscle	Electrode placement
Rectus femoris	Approximately midway between the anterior inferior iliac spine and the patella on the anterior side of the thigh
Vastus lateralis	Approximately two-thirds of the thigh length from the greater trochanter on the lateral side of the thigh
Vastus medialis	Approximately three-fourths of the thigh length from the anterior inferior iliac spine on the medial side of the thigh
Erector spinae	Three centimeters lateral to the L3 spinous process
Gluteus maximus	50% on the line between the sacral vertebrae and the greater trochanter. This position corresponds with the greatest prominence of the middle of the buttocks well above the visible bulge of the greater trochanter
Biceps femoris	Midway between the ischial tuberosity and the lateral condyle of the femur on the posterior side of the thigh
Semitendinosus	Midway between the ischial tuberosity and the medial condyle of the femur on the posterior side of the thigh
Reference electrode	The iliac crest of the right leg

**Table 2:** Repeated measures analysis of variance results of mean (±SD) EMG activity during squats performed with 80, 90, and 100% loads of 1RM as a percentage of maximal voluntary isometric contraction (% MVIC).

Muscles	80%	90%	100%
RF	36.1 ± 13.8	49.6 ± 34.3	52.3 ± 36.9
VM	56.9 ± 37.1	**67.4 ± 43.5** ^∗^	**73.6 ± 58.6** ^∗^
VL	53.6 ± 24.2	63.2 ± 37.8	67.7 ± 54.7
ES	40.8 ± 17.8	51.5 ± 25.1	53.8 ± 26.9
GM	27.8 ± 15.8	**34.1 ± 19.3** ^∗^	**38.7 ± 23** ^∗^
BF	23.5 ± 23.1	27.7 ± 25.3	30.4 ± 17.5
ST	21.5 ± 11.2	22.7 ± 15.7	28.2 ± 18.4

^∗^Repeated measures analysis of variance results significantly different (*p* < 0.05) from 80%.

**Table 3:** Repeated measures analysis of variance results of mean (±SD) EMG activity with 80, 90, and 100% loads of 1RM during descending and ascending phases of squat as a percentage of maximal voluntary isometric contraction (% MVIC).

Muscles	80%		90%		100%	
	Descend	Ascend	Descend	Ascend	Descend	Ascend
RF	35.8 ± 14	36 ± 16.2	51 ± 39.1	47.6 ± 26.9	53.8 ± 36.5	52.1 ± 38.3
VL	52.4 ± 20	59 ± 28.2	60.6 ± 40.8	65.7 ± 33.9	63 ± 40.9	72.4 ± 65.9
VM	52.8 ± 32.7	61.7 ± 43.8	**64.6 ± 42.8** ^∗^	70.4 ± 46.7	72 ± 57.6	**76.4 ± 61.8** ^∗^
ES	35.8 ± 14.4	41.9 ± 16.1	**50.1 ± 25.2** ^∗^	52.9 ± 25.2	**52.1 ± 27.2** ^∗^	55.8 ± 26
GM	19.7 ± 11.5	37.2 ± 20	**26 ± 15.7** ^∗^	44.8 ± 24.6	**30 ± 17.9** ^∗^	**50.2 ± 30.8** ^∗^
BF	19.6 ± 22.5	29 ± 24.8	22.1 ± 23.2	34.3 ± 28.3	23.8 ± 16.9	39.6 ± 23.1
ST	17 ± 10.4	29.1 ± 24	15.9 ± 7.2	29.1 ± 16.9	19.19 ± 11.4	39.5 ± 28.3

^∗^Repeated measures analysis of variance results significantly different (*p* < 0.05) from 80%.
